# Should we adjust erythropoiesis-stimulating agent dosage to postdialysis hemoglobin levels? A pilot study

**DOI:** 10.1186/1471-2369-13-60

**Published:** 2012-07-16

**Authors:** Nieves Castillo, Patricia García-García, Antonio Rivero, Alejandro Jiménez-Sosa, Manuel Macía, María Adela Getino, María Luisa Méndez, Javier García-Pérez, Juan F Navarro-González

**Affiliations:** 1 Nephrology Service, University Hospital Nuestra Señora de Candelaria, Santa Cruz de Tenerife, 38010, Spain; 2 MixedResearch Unit CHUC-ULL, Hospital Universitario de Canarias, La Laguna, Spain; 3 Research Unit, University Hospital Nuestra Señora de Candelaria, Santa Cruz de Tenerife, Spain

## Abstract

**Background:**

Predialysis hemoglobin (Hb) may overestimate the true erithropoiesis-stimulating agents (ESA) requeriments. We tested whether predialysis Hb is a reliable predictor of the postdialysis level to better control ESA dosage, and evaluated the relation between ESA, Hb and cardiovascular events (CVE).

**Methods:**

Cohort study including 67 stable hemodialysis patients. Pre- and post-dialysis Hb concentrations were measured, and ESA doses were calculated. A model to predict post-dialysis Hb is proposed. During 18 months follow-up, CVE, hospitalizations and mortality were collected.

**Results:**

After dialysis, Hb cocentration rise by 6.1 ± 5.6%. Using postdialysis Hb, the weight-adjusted ESA dosage would be lower respect to the prescription using predialysis Hb: 104 ± 120 vs 128 ± 124 U/kg/week (*P* < 0.001). Using predialysis Hb, 40.2% of subjects had a Hb level above 12 g/dL, whereas this percent increased to 70.1% using postdialysis Hb. During the follow-up, 15 patients had a CVE, without differences in Hb levels respect to subjects without CVE. However, patients with CVE had received higher ESA doses: 186 ± 180 vs 111 ± 98 U/Kg/week (*P* = 0.001). The prediction model is: Postdialysis Hb (g/dL) = 1.636 + 0.871 x predialysis Hb* (g/dL) + 0.099 x UF rate** (mL/kg/h) - 0.39 for women***. [R^2^ = 0.74; *P < 0,001; **P = 0.001; ***P = 0.03).

**Conclusions:**

Postdialysis Hb can be a better reflect of the real Hb level in hemodialysis patients. Using postdialysis Hb would avoid the use of inappropriately high ESA doses. The prediction of postdialysis Hb with an adjusted model would help us to identify those patients at risk for ESA overdosification.

## Background

The introduction of erythropoiesis-stimulating agents (ESA) has been the most important breakthrough in the treatment of anemia associated to chronic kidney disease (CKD). However, concomitantly with the growing knowledge about anemia management with ESA, new clinical challenges have emerged, such as the definition of the optimal hemoglobin (Hb) target for these patients. Moreover, this target has changed repeatedly based on security reasons [1-3]. Recent randomized clinical trials have shown more frequent adverse events in patients treated to higher Hb targets, and therefore receiving higher ESA doses [4-7]. This opens the question about the potential harmful effects of ESA, specially in high doses, and their potential contribution to adverse outcomes in these patients [8]. In addition, the potential association between ESA doses and adverse outcomes lead to question whether ESA dosage must be adjusted based on pre- or post-dialysis Hb values in hemodialysis (HD) population.

Clasically, prescription of ESA in HD patients has been based on the Hb concentration measured before the dialysis session, when volume overload may underestimate the real concentration of serum Hb. Diverse studies have described that serum Hb concentration varies during the HD session, and that Hb level is significantly different when it is measured before or after dialysis, or in the interdialysis period [9-12]. Therefore, predialysis Hb measurement might result in an overestimation of ESA dosage, representing a potential risk regarding ESA-related complications.

In the present study, we measured the Hb and Hematocrit (Hct) levels before and after a dialysis session in a group of stable maintenance HD patients in order to evaluate the variation of Hb and Hct during a dialysis session, the differences in EPO requirements and the associated economic impact. In addition, we prospectively analyzed the cardiovascular events (CVE) over a period of 18 months, and the potential relationship with Hb levels and EPO doses. Finally, since Hb measurement after the dialysis is not an usual clinical practice, we propose an equation to predict the post-dialysis Hb concentration based on an adjusted model.

## Methods

This cohort pilot study included 67 subjects in an outpatient hemodialysis centre at the University Hospital Nuestra Señora de Candelaria (Santa Cruz de Tenerife, Spain). Any research that is reported in this manuscript has been approved by the local Ethics Committee at the University Hospital Nuestra Señora de Candelaria, and has been carried out in compliance with the Helsinki Declaration. All patients gave informed consent to participate in the study.

All patients had CKD stage 5D, under maintenance HD for more than 6 months. There were 43 males and 24 females, with a mean age of 65 ± 13 years (range 31–85 years). All patients received bicarbonate hemodialysis three times weekly with polysulfone membranes (1.8-2 m^2^). The mean dialysis time was 237 ± 19 minutes (range 135–270 minutes). Regarding the underlying renal disease, 43% of patients had diabetic nephropathy. Sixty-one patients (91%) were under EPO therapy. There were no patients with hepatitis B, hepatitis C or human immunodeficiency virus infection. All subjects were on a stable clinical condition with no cardiovascular events in the previous 3 months. Baseline characteristics of the patients are shown on Table 1.

**Table 1:** Baseline characteristics of the patients

**Age(yr)**	**65 ± 13(range 31–85)**
**Sex** (male) —no. (%)	43 (64)
**Diabetes** —no. (%)	29 (43.2)
**Underlying disease** —no. (%)	
- Diabetic nephropathy	29 (43.2)
- Nephroangiosclerosis	15 (22.38)
- Glomerulonephritis	6 (8.95)
- Policystic kidney disease	8 (11.94)
- Other	9 (13.53)
**Charlson Comorbidity Index**	6.08 ± 2.18
**Time of dialysis** (minutes)	237 ± 19.58
**Kt-V**	1.28 ± 0.22
**Ultrafiltration** (ml/kg/h)	8.03 ± 2.98 (range 1.51-16.27)
**EPO dose per week** (U)	8,059 ± 7,712
**EPO dose per Kg and week** (U)	118 ± 120.24
**Type of EPO**—no. (%)	
- darbepoetin α	11 (16.4)
- epoetin α	16 (23.8)
- epoetin β	34 (50.7)
- without EPO	6 (9)

We measured the serum concentrations of Hb and Hct before and after the midweek HD session. We collected the prescribed EPO dose and calculated the dosage adjustment using the predialysis Hb level, as well as the hypothetical dose adjustment using the postdialysis Hb concentration. We calculated the difference and savings in dosage of EPO by patient and week, and the estimated cost reduction. The evolution of serum Hb concentration during an interdialysis period was analyzed in a small group of 5 patients. Serum Hb level was measured immediately before and after the dialysis session, and at 4, 24 and 48 hours postdialysis. In addition, we developed an adjusted model for the estimation of the postdialysis Hb concentration based on gender, predialysis Hb concentration and ultrafiltration (UF) rate.

During a follow-up period of 18 months, CVE, hospitalizations and mortality were recorded in order to analyze the potential relationship with Hb levels and EPO doses. The Charlson Comorbidity Index (CCI) [13], a score that includes age and comorbid conditions to which a specific weight is assigned, was used for valoration of comorbidity in each patient. Information was recorded by researchers from updated medical records. This index is used to adjust by comorbid conditions in longitudinal studies, and it has been specifically validated for patients with end-stage renal disease under dialysis therapy [14].

### Statistical analysis

Results for quantitative variables are expressed as mean and standard deviation. Results for categorical variables are expressed with frequencies and percentages. Proportions were compared using the Chi-square test. Within groups measures for continuous variables were compared with Wilcoxon test and between groups comparison were carried out using the Mann–Whitney U (asymptotic test) test and Wilkoxon-Mann–Whitney test (exact test). Differences in Hb serum concentration measured at predialysis, immediately postdialysis, and 4, 24 and 48 h after the end of the dialysis session were tested using the Wilcoxon rank test. Linear regression analysis was used to predict the postdialysis hemoglobin concentration using the predialysis hemoglobin level, the hourly UF rate (mL/kg/hour) and gender as the independent variables. Risk of CVE according to ESA dose was calculated using partial Cox regression analysis, and the results are expressed as Hazard Ratio and its 95% confidence interval. As only 15 cardiovascular events occurred, the Cox models presented are not cumulative, each used model is individual. Statistical analyses were performed with StatXact v. 5.0.3 (Cytel Co., MA), STATISTICA v. 7.1 (StatSoft, OK) and SPSS v. 17.0 (Chicago, IL). *P* values lower than 0.05 were considered statistically significant.

## Results

The mean predialysis and postdialysis Hb concentrations were 11.7 ± 1.1 g/dL and 12.5 ± 1.2 g/dL, respectively (*P* < 0.001). The mean intradialytic percent variation (%Δ) of Hb and Hct levels were 6.1 ± 5.6% (range: -6.5 to 17.27) and 5.8 ± 5.6% (range: -9.3 to 17.1), respectively. The mean weight loss during dialysis was 2.19 ± 0.79 kg (Range: 0.5 to 3.8 kg), with a mean hourly UF rate of 8.03 ± 2.98 mL/kg/h (range 1.51 to 16.27 mL/kg/h). When comparing patients according to the UF volume, in subjects with an UF > 2500 mL/session the mean %Δ of Hb and Hct were 9.2 ± 4.8% and 9 ± 4.4%, respectively, significantly higher than those observed in patients with an UF rate < 2500 mL/session: 3.9 ± 5.2% and 3.8 ± 5.3%, respectively (*P* < 0.001). Regarding the changes in serum Hb concentration during the interdialysis period, a statistical significant difference was found between the pre- and the immediately postdialysis Hb levels (*P =* 0.043). However, no differences were found between the immediately postdialysis Hb concentration and those at 4 h (*P* = 0.20), 24 h (*P* = 0.34) and 48 h (*P* = 0.08) after dialysis.

According to KDOQI guidelines [1], using the predialysis measurements, 37.3% of subjects had an adequate serum Hb concentration (11–12 g/dL), whereas 15 subjects (22.3%) had an inadequately low Hb level and 27 patients (40.2%) had a Hb concentration above 12 g/dL. However, when postdialysis determinations were used, 70.1% of patients had a serum Hb concentration inadequately elevated (Table 2). When postdialysis measurement was used, 6 out of the 15 patients (40%) with a predialysis Hb below 11 g/dL had a Hb concentration within the KDOQI target, whereas 16 out of the 25 patients (64%) with an adequate predialysis Hb level showed a postdialysis Hb concentration above 12 g/dL.

**Table 2:** Patients categorized according to pre and postdialysis Hb

**Hemoglobin (g/dL)**		**Postdialysis**		
		**Low (<11)**	**Normal (11-12)**	**High (>12)**
**Postdialysis**	**Low** (<11)	5	6	4
		7.5%	9.0%	6.0%
	**Normal** (11-12)	1	8	16
		1.5%	11.9%	23.9%
	**High** (>12)	0	0	27
		.0%	.0%	40.3%

Sixty-one patients (91%) were treated with EPO at the time of the study. No baseline differences were found between the patients with and without EPO in any variable (Table 3). We analysed the hypothetical dosage adjustment for EPO using the postdialysis Hb concentration as reference. If this determination was used, the weekly prescribed EPO dose would be significantly lower than the real EPO prescription based on predialysis Hb measurement (7,03 ± 7,765 vs 8,23 ± 8,045 U; *P* < 0.001). After adjusting for weight, the hypothetical EPO dosage using the postdialysis Hb concentration could be reduced by 18.7% (EPO dosage using predialysis Hb, 128 ± 124 U/kg/week; EPO dosage using postdialysis Hb, 104 ± 120 U/kg/week).

**Table 3:** Baseline comorbidities categorized by ESA dose

**Comorbidity - N (%)**	**ESA dose (>18000 IU/week) N = 7**	**ESA dose (≤18000 IU/week) N = 60**	** *P* **
**Diabetes**	3 (42.9)	26 (43.3)	0.99
**Ischemic Heart Disease**	1 (14.4)	20 (33.3)	0.41
**Heart failure**	1 (14.3)	11 (18.3)	0.99
**Peripheral vascular disease**	3 (42.9)	12 (20)	0.18
**Stroke**	0 (0)	7 (11.7)	0.99
**Chronic Obstructive**			
**Pulmonary Disease (COPD)**	1 (14.3)	16 (26.7)	0.67
**Arrhytmias**	2 (28.6)	11 (18.3)	0.61
**Tumoral History**	2 (28.6)	7 (11.7)	0.24
**Hepatopathy**	1 (14.3)	3 (5)	0.36
**Hypertension**	7 (100)	58 (96.7)	0.99

The linear regression model to predict the postdialysis Hb concentration is as follows: Postdialysis Hb (g/dL) = 1.636 + 0.871 x predialysis Hb* (g/dL) + 0.099 x UF rate**(mL/kg/h) - 0.39 for women***. The adjusted coefficient of determination (R^2^) was 0.74 (*predialysis Hb, *P* < 0.001; **UFR, *P* = 0.001; ***gender, *P* = 0.031).

During the 18 months follow-up, 15 (25%) patients had a CVE (2 stroke, 4 coronary disease, 2 cardiac arrhythmia, 1 peripheral vascular disease, 4 arteriovenous fistula thrombosis and 2 mesenteric ischemia). No differences were found between patients with and without CVE on Hb levels (11.2 ± 1.43 mg/dL vs 11.9 ± 1.02 mg/dL, respectively; *P* = 0.14). However, patients who had a CVE had received higher mean doses of EPO than those without events: 12,133 ± 10,562 U/week vs 7,615 ± 6,963 U/week; *P =* 0.037. Adjusted by weight the differences were 186 ± 180 vs 111 ± 98 U/Kg/week (*P* = 0.001). None of the 6 patients without EPO therapy presented any CVE. We investigated whether comorbidities, expressed as Charlson index, or adequate dialysis (as measured by Kt/V), were confounding factors associated with CVE and EPO dose. No association was found neither between Charlson index and CVE (r = 0.11; P = 0.19), nor EPO dose (r = 0.04; *P* = 0.37). Regarding Kt/V, this parameter was similar in patients with and without CVE (1.3 ± 0.19 vs 1.3 ± 0.23, respectively), and no association was found between Kt/V and EPO dose (r = 0.19; *P* = 0.58). Partial Cox regression analysis showed that controlling for ischemic heart disease and heart failure, ESA dose is a risk factor for CVE. However, the confounding effect of age, arrthymia, diabetes, peripheral vascular disease and stroke is unclear (Table 4). Frequency of hospitalization was significantly higher in patients with CVE. Likewise, length of stay was also higher in patients with CVE (6 days [range, 3 to 12]) than in those without CVE (0 days [range, 0 to 7]); *P* = 0.016). A total of 13 deaths occurred during follow-up, with an increase in the probability of death in patients with CVE (53% vs 9.63%; *P* <0.001).

**Table 4:** Partial Cox regression models to predict CVE controlling by age and comorbidities

	**HR**	**95% CI**	** *P* **
** *Model 1* **			
**EPO dose** (IU/week)	1.015	1.010-1.020	0.032
**Ischemic heart disease**	2.66	0.920-7.650	0.069
** *Model 2* **			
**EPO dose** (IU/week)	1.0015	1.001-1.002	0.049
**Heart failure**	2.29	0.710-7.270	0.16
** *Model 3* **			
**EPO dose** (IU/week)	1.00	0.999-1.001	0.058
**Stroke**	1.72	0.380-7.750	0.47
** *Model 4* **			
**EPO dose** (IU/week)	1.00	0.999-1.001	0.06
**Age** (years)	1.005	0.967-1.045	0.79
** *Model 5* **			
**EPO dose** (IU/week)	1.000	0.999-1.001	0.065
**Arrythmia**	1.17	0.330-4.170	0.80
** *Model 6* **			
**EPO dose** (IU/week)	1.000	0.999-1.001	0.094
**Diabetes**	0.468	0.163-1.345	0.159
** *Model 7* **			
**EPO dose (IU/week)**	1.000	0.999-1.001	0.105
**Peripheral vascular disease**	1.702	0.539-5.372	0.36

Finally, the aplication of postdialysis Hb to calculate the EPO dosage was associated with a reduction in cost: 101 ± 111 vs 124 ± 116 euros/patient/week, respectively (*P* < 0.001). In our dialysis center, for the 67 patients included in the study, this action would have saved 83,214 euros ($ 118,858)/year.

## Discussion

The main finding of our study is that the use of postdialysis Hb concentration to monitor and adjust EPO dosage would result in a significant reduction of EPO requeriments and costs. Specifically, this action in the present study was associated with a mean EPO dose decrease of 18.7% (U/Kg/week), and a mean cost reduction of 1,242 euros/patient/year ($ 1,774/patient/year). In addition, we present a simple model to estimate the postdialysis Hb level based on predialysis Hb, UF rate and gender. Our suggestion about the use of postdialysis Hb level to adjust and monitor ESA dosage may be a relevant issue, specially at the present moment, when the emergence of safety concerns and the changes in clinical practice recommendations have influenced physician practice in terms of reduction of target Hb, use of ESA and dosing regimens [15].

The Food and Drug Administration has recently announced that ESA should be prescribed under a risk management programme (the Risk Evaluation and Mitigation Strategy), since results from three recent randomized controlled trials – the Correction of Hemoglobin and Outcomes in Renal Insufficiency (CHOIR) [4], the Cardiovascular Risk Reduction by Early Anemia Treatment with Epoetin Beta (CREATE) [5], and the Trial to Reduce Cardiovascular Events with Aranesp Therapy (TREAT) [6] – showed that assigned chronic kidney disease patients (not in dialysis) to intervention with an ESA to achieve a high versus a low Hb target was associated with an increased risk of myocardial infarction, stroke and death. However, almost 10 years before, the Normal Hematocrit Trial (NHCT) [16], a study in which more than 1200 HD patients with congestive heart failure or ischemic heart disease were randomized to target Hct values of 42% (normal Hct group) and 30%, had to be stopped prematurely by the Data Safety Monitoring Board because of concerns of increased cardiovascular disease risk and mortality in the normal Hct arm.

Although the majority of studies related to anemia in HD have used predialysis Hb and Hct values, several works have analysed the postdialysis values of Hb, showing important results. Vlassopoulos et al. [9] measured Hb and Hct in 15 stable patients before the HD sesion, and 24, 48 and 72 hours in the interdialysis period. They observed a significant 24 h postdialysis rise in Hb and Hct as compared to the predialysis level, with a gradual decrease reaching non-significant differences before the next HD session. Similar findings were reported by Movilli et al. [10] and Bellizzi et al. [12]. These authors observed that Hb and Hct levels after dialysis were similar to those observed immediately at the end of the HD session, and that these concentrations were significantly higher than the predialysis ones. Similarly, a significant increment in Hb and Hct levels after the HD procedure was observed in our study. The mean percent increase in the Hb and Hct values were 6.1% and 5.8%, respectively, and rise to around 9% in patients with a body weight loss ≥ 2.5 kg/session. Moreover, serum Hb concentrations measured at 4, 24 and 48 hours postdialysis remained elevated as compared with the predialysis Hb measurement, without significant differences respect to the immediately postdialysis Hb level. According to KDOQI guidelines [1], using postdialysis measurements, 70.1% of our patients had a serum Hb above 12 g/dL. Importantly, this represents that 64% of patients with a normal Hb target based on predialysis Hb concentration had an inadequately high Hb level when postdialysis determinations were used. These findings indicate that during the first 24–48 hours after the HD session a slow reequilibration occurs, first affecting to the extravascular space, and then to the intravascular compartment [10,12], suggesting that HD patients present most of the time a Hb and Hct levels clearly higher (up to 15%) than the predialysis values [9]. This results in the potential exposition of these patients to excessively high Hb and Hct concentrations for a prolonged period.

During the 18 months of follow-up in our study, 25% of patients had a CVE. There were no differences regarding Hb levels between patients with and without CVE. Conversely, patients who had a CVE had received significantly higher EPO doses than those without events. This relationship was not affected by some baseline comorbidities or dialysis adequacy, but probably the small sample size makes unclear the effect of other (i.e. diabetes, age, arrthymias, peripheral vascular disease and stroke). In addition, no CVE were observed in the 6 patients without ESA therapy. This observation points to the potential adverse outcomes associated with ESA administration, which has been suggested by other studies. Using data from the United States Renal Data System administrative claims, a retrospective cohort study of more than 90,000 prevalent HD patients showed a significant nonlinear relationship between increased epoetin dose and mortality [17]. In another study that comprised more than 40,000 maintenance HD patients, an association between erythropoietin exposure and mortality over 3 years was observed at very high doses (> 20,000 units/week) [18]. To further analyze the relationship among patient comorbidity, ESA dosage, and evolution with death hospitalization, myocardial infarction, stroke and heart failure as the primary compound objective, a secondary analysis of the CHOIR study was recently performed [8]. In the adjusted models, the risk associated with randomization to the high-Hb group was not significant (p = 0.49), whereas high-dose erythropoietin dosage was associated with a 57% higher risk of the main variable (HR 1.57; CI: 1.04 to 2.36, p = 0.03). Therefore, although the results of these studies did not prove causality, they suggested that high ESA dose rather than the Hb level was associated with adverse outcome, reinforcing the actual recommendations about the strict adjustment of ESA dosage and the close monitorization of Hb levels.

## Conclusion

In conclusion, our results highlight the potential importance of postdialysis Hb concentration to monitor the Hb and Hct target levels, and to adequately adjust the ESA dosing regimen. The use of postdialysis Hb would avoid exposure of patients to inadequately high Hb and Hct levels during the interdialysis period, as well as to excessive ESA doses. This may be especially relevant in the current scenario: the optimal Hb target is not yet established, with serious safety concerns regarding ESA therapy. Since the measurement of postdialysis Hb concentration might be an unfeasible practice in many HD units, we propose an adjusted model to predict this parameter. The prediction of postdialysis Hb with an adjusted model help us to identify those patients at risk for overdosification of ESA, and might be associated with beneficial effects regarding cardiovascular morbidity and mortality. In addition, this strategy may result in lower ESA requeriments, or even may lead to the avoidance of ESA therapy in several patients, with a beneficial impact on the economic cost. From this economical perspective, the estimation of ESA dosage based on postdialysis Hb concentration would represent a mean saving of 18.5%, approximately 1,242 euros ($ 1,774/patient/year).

## Competing interests

The authors declare that they have no competing interests.

## Authors’ contributions

NC, PGG, JGP, JFNG have participated on the protocol designed, coordination of the study, analysis of data, and drafted the manuscript. AR, MM, MAG, MLM participate in data collection and interpretation, and preparation of the manuscript after the draft suggested by the previous authors. AJS, NC and PGG performed the statistical analysis. NC, PGG and JFNG participated in the corrections of the final draft of the manuscript. All authors have read and approved the final version of the manuscript.

## Pre-publication history

The pre-publication history for this paper can be accessed here:

http://www.biomedcentral.com/1471-2369/13/60/prepub
